# Repeated introductions and widespread transmission of human metapneumovirus in Côte d’Ivoire

**DOI:** 10.1186/s12879-025-11512-2

**Published:** 2025-09-02

**Authors:** Hervé A. Kadjo, Sairah M. Khan, Sana Tamim, Meriadeg Ar Gouilh, Marius Adagba, Edgard Adjogoua, Daouda Coulibaly, Astrid Vabret, Joshua L. Cherry, Martha I. Nelson, Nídia S. Trovão

**Affiliations:** 1 Department of Epidemic Viruses, Pasteur Institute of Côte d’Ivoire, Abidjan, Côte d’Ivoire; 2 Division of International Epidemiology and Population Studies, Fogarty International Center, National Institutes of Health, Bethesda, MD 20892 USA; 3 Department of Virology/Immunology, National Institute of Health, Park Road, Chak Shahzad, Islamabad, Pakistan; 4 Department of Virology, University of Caen Normandy, Dynamicure INSERM UMR 1311, Centre Hospital-Universitaire (CHU) Caen, Caen, France; 5 Department of Epidemiology and Disease Surveillance, National Institute of Public Hygiene, Abidjan, Côte d’Ivoire; 6 Division of Intramural Research, National Library of Medicine, National Institutes of Health, Bethesda, MD USA

**Keywords:** Human metapneumovirus, Evolution, Disease severity, Phylodynamics, Acute respiratory infections

## Abstract

In Cote d’Ivoire, the incidence rate of acute respiratory infections (ARIs) rose from 165 cases per 1000 children in 2014 to more than 200 cases per 1000 children in 2015. The genetic diversity, transmission dynamics, and epidemiology of human metapneumovirus (hMPV), a causative agent of ARIs, in Cote d’Ivoire are unknown. This information is key in comprehending the transmission patterns and the role of global strains in establishing local epidemics in the country.

Demographic information and biological samples were collected from 3,899 children under five-years-old, from January 1, 2013 to December 31, 2015 through Côte d’Ivoire’s Influenza surveillance network. Phylodynamic modeling was performed on sequences of the surface and attachment glycoprotein genes (F and G, respectively).

A total of 6.23% (*n* = 243/3899) of the samples were positive for hMPV. We observed continuous transmission of hMPV in Côte d’Ivoire throughout the year with peaks in the two dry periods from February to March and July to September. Phylodynamic modeling revealed the circulation of the two large groups of genotypes A and B as well as lineages A, B, B1, and B2. Viral introductions into Côte d’Ivoire were estimated to have occurred 2011–2015 for the F gene genotypes and 2007–2014 for G gene genotypes. Through phylogeographic modeling, we estimated at least 14 viral introductions into Côte d’Ivoire during this period frequently from regions with available sequence data (e.g., Asia).

Molecular surveillance and characterization of the evolutionary mechanisms and the spread of hMPV in Côte d’Ivoire allows for differentiating the burden caused by this virus and other co-circulating respiratory viruses like RSV and influenza. Our findings may inform potential vaccine designs for hMPV similar to the recent success for RSV. Therefore, larger-scale and continuous genomic and epidemiological surveillance of hMPV globally and in Côte d’Ivoire is essential for identifying viral introductions and implementing control strategies.

**Supplementary Information:**

The online version contains supplementary material available at 10.1186/s12879-025-11512-2.

## Introduction

Acute respiratory infections (ARIs) are a leading cause of morbidity and mortality worldwide, particularly in children under five in developing countries [1]. In 2008, lower respiratory tract infections caused an estimated 1.9 million deaths in this age group, with over 70% occurring in Sub-Saharan Africa and Southeast Asia [2]. The disproportionate risk of pneumonia in developing countries [3] highlights why ARIs remain a major public health challenge.

Recent world events such as the outbreak of the SARS Coronavirus epidemic in 2003, the transmission of the avian A (H5N1) virus to humans in 2005 and the outbreak of the Influenza A (H1N1) pdm09 in 2009, COVID-19 pandemic in 2020, alerted the world to the danger of viruses in a globalized world where a dangerous pathogen can emerge and spread to all continents within days. The large number of viruses in animals and the changing dynamics of the human-animal interface guarantees the emergence of new respiratory pathogens [4]. In addition, the efficient transmission of respiratory viruses from person to person makes their control very difficult. The best hope for effectively responding to emerging threats is to improve the management of viral respiratory infections and their sequelae. Controlling the morbidity and mortality of ARI of viral origin is based on a better understanding of the epidemiology of the disease, the availability of adequate laboratory diagnostic capacity and the accessibility of antivirals. With the advancement of molecular diagnostic techniques in recent years, access to the diagnosis of viral respiratory infections has been improved [5, 6]. However, these techniques are still limited to specialized laboratories, resulting in an underestimation of the viral etiology in cases of ARI.

Among the viral etiological agents responsible for ARI, hMPV was first described in 2001 from children in Netherlands [7]. hMPV is a negative-sense single-stranded RNA virus belonging to the Metapneumovirus genus, of the *Pneumovirinae* subfamily, from the *Paramyxoviridae* family [8]. The viral genome contains eight genes, of which the surface F and G genes play important roles in the epidemiology and pathogenesis of infection [9, 10]. Upon its description, at least two genetic lineages of hMPV co-circulated in humans [7]. Phylogenetic analysis of additional sequences obtained for the F and G genes revealed that each of these main genotypes, A and B, could be further divided into lineages: A1, A2 and B1, B2 [7, 11]. hMPV is one of the main infectious pathogens responsible for acute respiratory illness in humans worldwide. Since its initial description, hMPV has been reported worldwide and children under 2–5 years are the most commonly affected age group. It is implicated in the onset of infant bronchiolitis [12]. It primarily infects children but affects all age groups worldwide.

In Côte d’Ivoire, ARIs rank third in terms of morbidity and mortality among children under 5, after diarrheal diseases and those of the expanded immunization program (EPI). From an epidemiological point of view, although respiratory infections in children are a problem, data on the viral etiology of ARI and their burden at the population level are limited or non-existent [13]. ARIs caused the death of more than 11,000 children in 2012, and according to figures from the Annual Health Situation Report (RASS) from 2014 and 2015, ARIs incidence rate increased, with estimates of 202 cases per 1000 children in 2015 compared to 165 cases per 1000 children in 2014. The global threat posed by the influenza A (H1N1) pdm09 virus has spurred efforts to improve surveillance and monitoring systems. These efforts, implemented since 2007, have improved the understanding of the epidemiology of influenza and to determine the epidemiological characteristics of the virus in Côte d’Ivoire [14]. In 2013, a study was carried out to confirm the circulation of respiratory viruses other than influenza. The study in Côte d’Ivoire identified, in order of frequency, human Rhinoviruses hRhV (31.92%), respiratory syncytial virus RSV (24.75%), Parainfluenza virus PIV (20.5%), human coronavirus HCoV229E (12.05%), hMPV (6.2%), human coronavirus HCoVOC43 (1.0%) and Enterovirus EnV (1.0%) [15]. To date, little data is available to illuminate the epidemiology of acute respiratory infections related to hMPV, and no molecular data to unveil its genetic diversity and transmission dynamics in the country. This study substantially increases the number of hMPV sequences from West Africa and aims to address the lack of knowledge of hMPV molecular evolution by reconstructing its evolutionary history and transmission dynamics in Côte d’Ivoire. This work sheds light on hMPV transmission (seasonality, spread patterns) to inform public health strategies to assist in improving the control of hMPV and enhanced understanding of hMPV genetic diversity in Côte d’Ivoire for the development of vaccines and antiviral drugs against hMPV infection.

## Material and methods

### Study design

This is a descriptive, cross-sectional study, conducted at the Respiratory Viruses Unit of the Institute Pasteur of Côte d’Ivoire. This unit houses the World Health Organization (WHO) National Reference Laboratory for influenza and other respiratory viruses. We analyzed the epidemiological data and biological samples from the national sentinel influenza surveillance network. The network was built according to WHO guidelines [16] and consists of nine health centers located in health areas covering a population of approximately 1.9 million inhabitants. These sites are located in several geographical areas of the country (Supplementary Fig. 1), and data was collected during the period between January 1 st, 2013 and December 31 st, 2015.

### Study population

Each sentinel site recruited five patients per week, who met the influenza-like illness (ILI) or severe acute respiratory infection (SARI) case definition and were 5 years of age or younger and whose guardians gave consent for the survey. All subjects with fever (temperature ≥ 38 °C) and coughing for less than 10 days were admitted as ILI cases. SARI was evoked in all subjects feeling feverish or with fever and cough, or breathing difficulties that had been going on for less than 10 days and whose condition required hospitalization. We excluded from the study children with a real-time reverse transcription-polymerase chain reaction (RT-PCR) result positive for influenza virus and RSV.

### Sample and environmental data collection

A total of 3**,**899 nasopharyngeal swabs were collected using the Copan Universal transport medium (UTM-RT) system. The samples were obtained during consultation or hospitalization, and were immediately stored in a cooler with a cold accumulator or a refrigerator at + 4 °C before their transfer to the reference laboratory at the Institute Pasteur. An epidemiological fact sheet with demographic data, patient history, signs of severity, and risk factors for influenza infection was completed for all subjects included in the study, as samples were obtained as part of the national influenza sentinel surveillance framework. For each site, climate data (temperature, humidity**,** and rainfall) were collected from the Société d’Exploitation et de Développement Aéroportuaire, Aéronautique et Météorologique (SODEXAM) in order to determine the correlation between positive cases of hMPV and these different climatic parameters.

### Statistical analysis

Data management and analysis were performed using R software (version 2.15.1) after initial data entry in Epi-info® (version 3.5). Descriptive statistics were used to summarize the data; quantitative variables were described by their medians, while categorical variables were presented as counts and proportions.

To analyze associations between categorical variables (e.g., hMPV positivity by sex, age group, or clinical presentation), the chi-square (χ^2^) test or Fisher’s exact test was used as appropriate. The relationship between monthly hMPV case counts and climatological factors (temperature, humidity, rainfall) was assessed using Spearman’s correlation and a multiple linear regression model. A *p*-value < 0.05 was considered statistically significant for all tests.

### RNA extraction and real time RT-PCR for hMPV detection

RNA was extracted from 200 μL of the collected nasopharyngeal secretion in Universal Transport Medium (UTM-RT). Extraction was performed using the QIAamp Viral RNA Mini kit (QIAGEN®, Hilden, Germany) as per the manufacturer’s instructions with RNA elution into a final volume of 80 μL of AVE buffer. Nuclease-free water was included for each extraction run as a negative control. Two aliquots of each extracted RNA sample were made, one of the aliquots was used for RT-PCR targeting the N gene of hMPV, and the second was stored at −20°C for future analyses.

The master-mix for RT-PCR was made with the Invitrogen™ SuperScript™ III Platinum™ Kit One-step quantitative RT-PCR System with the following primers and probes:

- Fwd-5’-ATGTCTCTTCAAGGGATTCACCT-3’,
- Rev-5’-AMAGYGTTATTTCTTGTTGCAATGATGA-3’,
- Pr-(JOE) -5’-CATGCTATATTAAAAGAGTCTCARTAC-(BHQ-1) -3’.

The cycling conditions were 30 min (min) at 50 °C, 2 min at 95 °C, 45 cycles of 15 s (s) at 95 °C, 15 s at 55 °C, and 15 s at 55 °C; and a final incubation at 55 °C for 10 min.

### Amplification and sequencing of the F and G genes

Conventional RT-PCR was also used to amplify a fragment of the F and G gene open reading frames (ORFs) of 243 positive samples obtained by real time PCR, from hMPV samples. The amplification RT-PCR was performed using the Qiagen One-Step RT-PCR kit (Qiagen) as per the manufacturers’ instructions, using the following primer sequences:

- F gene Fwd-5’-CAATGCAGGTATAACACCAGCAATATC-3’,
- F gene Rev-5^’-^GCAACAATTGAACTGATCTTCAGGAAAC-3’ [17].
- G gene Fwd-5’-GAGAACATTCGRRCRATAGAYATG-3’ [18].
- G gene Rev-5’-AGATAGACATTRACAGTGGATTCA-3’.

Thermocycling was performed following reverse transcription at 50 °C for 30 min, PCR activation at 94 °C for 15 min, 40 cycles of denaturation at 94 °C for 30 s, annealing at 55 °C for 1 min, extension at 72 °C for 1 min, followed by a final extension at 72 °C for 10 min. All PCR products were visualized using the flashgel system of Lonza® with 1.5% agarose gel. Sequencing was performed in both directions using an ABI 3500 XL Genetic Analyzer with BigDye terminators (Applied Biosystems) at a contract sequencing facility (Genewiz sequencing, Germany). The complete genome sequences of the reference prototypes of each lineage were available in GenBank (under the accession numbers AF371337 [A1], FJ168779 [A2], AY525843 [B1] and FJ168778 [B2]).

### Dataset assembly

In order to supplement the Côte d’Ivoire hMPV datasets of 20 F gene lineage A, 43 F gene lineage B, 21 G gene lineage A, and 29 G gene lineage B sequences, we retrieved all available hMPV sequences from GenBank on September 6, 2019, that were annotated with location and collection date. This initial background dataset consisted of 2,345 F and 1,554 G sequences.

The sequence were aligned using MAFFT v7.409 [19] and subsequently manually inspected in AliView v1.25. During manual editing, one Côte d’Ivoire sequence was removed from the G gene dataset because of a duplication not seen in other Côte d’Ivoire or background sequences. We also removed 145 nucleotides (nt) from G2403 and G357 in the G: B2 dataset out of concern that the 145-nt were the result of recombination [20].

We noted the 180-nt and 111-nt duplications in G gene samples reported by Saikusa et al. [21] and preserved them in the initial G gene alignments. Any background sequences that were noted as nonfunctional in their GenBank entries or had premature stop codons that would likely impair function were also removed.

Because the Côte d’Ivoire sequences are fragments, we trimmed the total dataset of each gene to match the length of the Côte d’Ivoire sequences. The F dataset was trimmed to 686-nt positions and the G dataset to 868-nt positions. After each dataset was trimmed, we removed samples shorter than 75% of the length of the longest sequence.

After editing the alignments, maximum-likelihood (ML) trees were inferred using RAxML v7.2.6. [22] incorporating a general time reversible (GTR) model of nucleotide substitution with a gamma-distributed (Γ) rate variation among sites. We investigated the temporal signal of the datasets using TempEst [23]. A few background sequences showing incongruent temporal patterns were excluded (Supplementary Fig. 2). In order to confidently identify clades within each gene, each ML tree was run with 5,000 bootstrap replicates. We extracted clades encompassing the Côte d’Ivoire samples at nodes with high bootstrap support (> 70%). In the F gene ML tree, we identified three clades of interest (A, B1, B2; Supplementary Fig. 3) and in the G gene ML tree, four clades (A, B Early, B1, and B2) were selected. The “B Early” clade was designated to describe a phylogenetically distinct group of G gene sequences that could not be classified within the canonical B1 or B2 clades. As shown in the phylogenetic tree (Supplementary Figure S4), this clade occupies a basal position relative to the other B lineages, meaning it diverges from the main trunk of the B genotype before the common ancestor of the B1 and B2 clades. The name “B Early” was therefore chosen to reflect this early-diverging characteristic. We extracted the sequences for each new tree to create “gene: clade” specific datasets, which were subsequently aligned and manually edited. Duplicate sequences with identical countries, collection dates, and nucleotide sequences were removed (Supplementary Figs. 5–8). Specific to the G: A dataset, the region caused by the 180-nt insertion, encompassing the 111-nt insertion, was removed. We used RAxML to generate “gene: clade” ML trees which were examined in TempEst for outliers (see Fig. 2 below).

### Phylogenetic analysis

To reconstruct the evolutionary history and spatial diffusion of the virus, we employed a Bayesian phylogenetic and phylogeographic framework for each of the “gene: clade” datasets separately using the high-performance computational capabilities of the Biowulf Linux cluster at the National Institutes of Health, Bethesda, MD, USA (http://biowulf.nih.gov). Evolutionary rate variation across lineages was accounted for using an uncorrelated relaxed molecular clock model with branch rates drawn from a log-normal distribution. Changes in the viral effective population size (Ne) over time were inferred using a Skygrid demographic prior [24]. Nucleotide substitution was modeled using a general time reversible (GTR) model with gamma-distributed rate variation among sites. For sequences where only the year of collection was available, tip dates were accommodated by uniform sampling within a one-year window (January 1 st to December 31 st).

Markov chain Monte Carlo (MCMC) analyses were conducted using BEAST v1.10.4, with computational performance enhanced by the BEAGLE library [25]. Each dataset was analyzed in at least three independent MCMC runs of 400 million iterations, with sampling every 40,000 iterations. Convergence of all parameters was assessed visually using Tracer v1.7.1, and statistical uncertainty was quantified using 95% highest posterior density (HPD) intervals. A burn-in of at least 10% was applied to each chain.

Given the independent modeling of the diffusion and substitution processes, we adopted a two-step inference approach. First, we focused on the sequence evolution process to generate an empirical distribution of phylogenetic trees. Subsequently, this distribution of trees was used as a condition for inferring the discrete location diffusion process. A subset of 500 trees was randomly selected from the combined posterior distribution of trees to represent the empirical distribution for spatial diffusion analysis [26].

Spatial diffusion dynamics among specified countries were estimated for each “gene: clade” dataset using a Bayesian discrete phylogeographic approach [27]. This approach models location transitions as a continuous-time Markov chain (CTMC) process, allowing for the inference of ancestral location states. A nonreversible CTMC model [28] was employed, and Bayesian stochastic search variable selection (BSSVS) was incorporated to identify a sparse set of significant transition rates, representing epidemiological connectivity [29]. Furthermore, Markov jump histories for location traits were mapped across the posterior tree distribution using stochastic mapping techniques [30], and the number of jumps was summarized.

Maximum clade credibility (MCC) trees were generated using Tree Annotator v1.10.4, and visualizations were produced using FigTree v1.4.3.

## Results

### Population, sex and age distribution

A total of 3,899 children under five years of age were recruited between January 1, 2013 and December 31, 2015. The study included 2062 males and 1837 females, resulting in a male-to-female ratio of approximately 1.12:1. The 12- to 24-month age group was the most represented with 38.88% (1516/3899) of recruitments. The median age of study participants was 12 months. Of the children recruited, 80.12% (3124/3899) had an influenza-like illness and 19.87% (775/3899) had severe acute respiratory infection. The positivity rate for hMPV infection was 6.23% (243/3899). There was no statistically significant association between the detection of hMPV and sex (*p* = 0.466), age group (*p* = 0.145), or clinical presentation (*p*-value = 0.475) (see Table 1).

**Table 1:** Distribution of hMPV cases by sex, age group, and clinical presentation (2013–2015)

Variables	Categories	Total cases tested	Positives (%)	Negatives (%)	Chi^2^ (*p*-value)
Sex	Male	2062 (100)	134 (6.4)	1928 (93.6)	*p* = 0.466
	Female	1837 (100)	109 (5.9)	1728 (93.9)	
	Total	3899 (100)	243 (6.23)	3656 (93.77)	
Age groups (months)	0–11	1399	87 (6.2)	1312 (93.8)	*p* = 0.145
	12–24	1516	110 (7.23)	1406 (92.77)	
	25–36	468	22 (4.7)	446 (95.3)	
	37–48	291	14 (4.8)	277 (95.2)	
	> 48	225	10 (4.4)	215 (95.6)	
	Total	3899	243 (6.23)	3656 (93.77)	
Clinical symptoms	ILI	3124	199 (6.37)	2925 (93.63)	*p* = 0.475
	SARI	775	44 (5.7)	731 (94.3)	

*ILI* influenza like illness, *SARI* Severe acute respiratory infection

### Seasonality and correlation between climatic factors and the circulation of hMPV

**Fig. 1:**
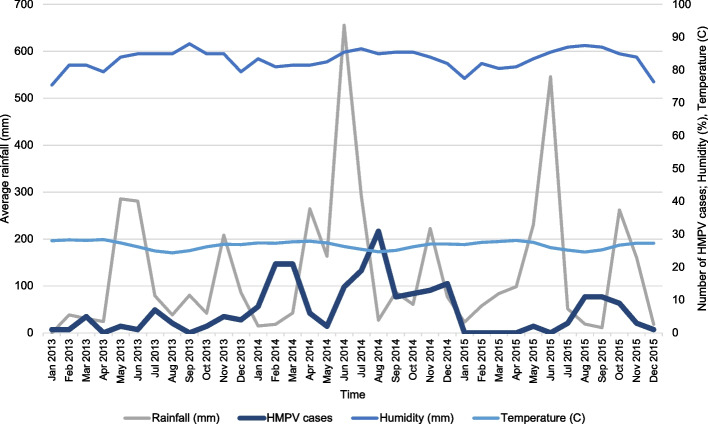
Monthly epidemiological trend curve of hMPV cases in relation to average percent humidity, average temperature in Celsius, and average rainfall (mm) in Abidjan and San-Pedro for the period of 2013–2015

Positive cases of hMPV were detected every month during the study period (January 2013 to December 2015). Of the 243 positive hMPV cases, 80.24% (195/243) originated from sentinel sites in Abidjan and San Pedro. These coastal cities experience a humid equatorial climate with abundant rainfall, consistent relative humidity, and minimal temperature variation. Climatic data (temperature, humidity, rainfall) were available for these locations. Analysis revealed two circulation peaks: one from February to March and another from July to September, coinciding with the country’s two dry seasons. While the monthly incidence of hMPV did not directly correlate with average ambient temperature or humidity. An increase in hMPV cases was observed with decreases in mean rainfall levels (Fig. 1). However, Pearson correlation tests showed no statistically significant association at the 5% level between monthly hMPV incidence and mean temperature, humidity, or rainfall. Furthermore, multiple linear regression analysis indicated no significant correlation (R^2^ adjusted = 0.02, *p* = 0.30) between monthly hMPV cases and these combined climatic parameters.

### Genetic diversity and evolutionary reconstruction

**Fig. 2:**
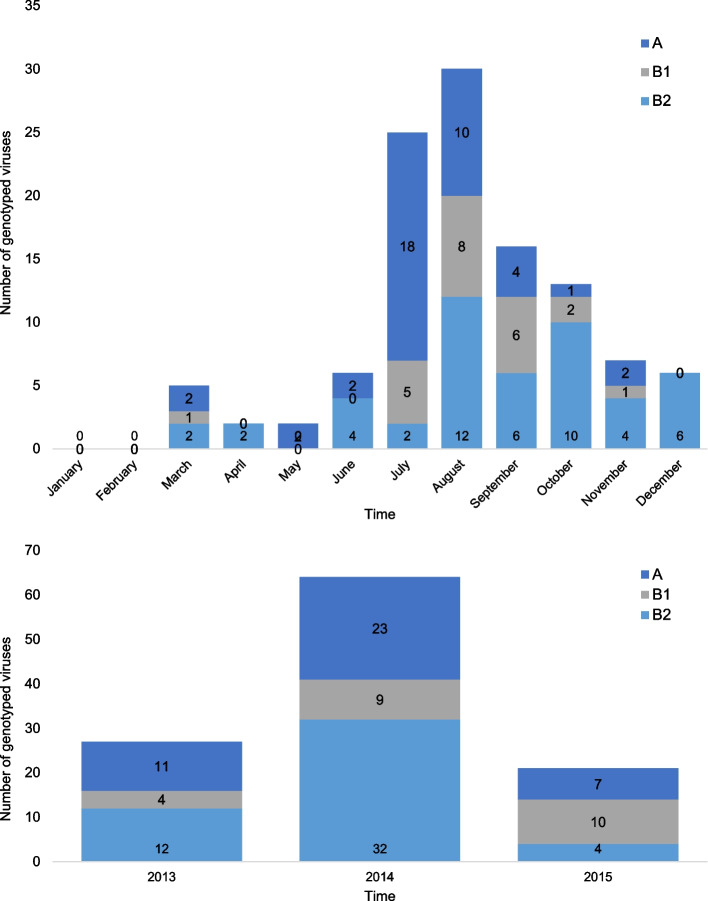
Monthly distribution of circulating hMPV genotypes for the period of 2013–2015

Among the 173 samples that were sent for sequencing, the final dataset comprised of 63 F gene sequences and 50 G gene sequences from the Côte d’Ivoire pediatric cohort during 2013–2015. These sequences belonged to different genetic lineages. For the F gene, they were evenly characterized as lineages A, B1 and B2. For the G gene, the majority of sequences belonged to lineages B2 and A, with only a few clustering within the B1 and B Early clades (Supplementary Table S1, Supplementary Figs. 2 and 3). Among the characterized strains, the majority of lineage B (87.5%, 63/72) and lineage A (92.7%, 38/41) were associated with influenza-like illness (ILI). Statistical analysis revealed no significant association between viral genotypes and clinical presentation (*P* = 0.615). The monthly distribution of genotypes showed that genotype A was frequently detected between March and August, with a peak in July suggesting circulation during the rainy season. Genotype B1 was detected from April to October, peaking in August, while genotype B2 showed a more prolonged distribution from June to December (Fig. 2, top panel). Analysis of the annual distribution revealed dynamic shifts in genotype prevalence during the study period (Fig. 2, bottom panel). In 2013, genotypes A and B2 co-circulated at similar levels. This was followed by a large epidemic season in 2014 that was overwhelmingly dominated by genotype B2. In 2015, circulation shifted again, with genotype B1 emerging as a major co-circulating lineage alongside genotype A, while detections of B2 decreased substantially.

**Fig. 3:**
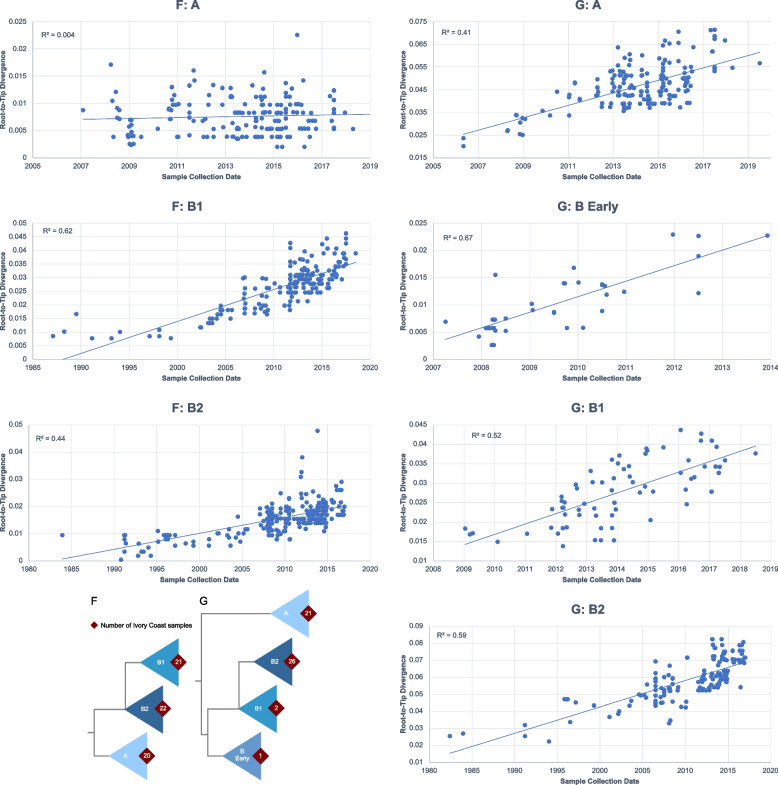
Root-to-tip divergence as a function of sampling time for maximum-likelihood tree of hMPV’s F and G genes. Left panels represent estimates for F gene’s A clade, B1 clade, and B2 clade, top to bottom, respectively. Right panels represent estimates for G gene’s A clade, B Early clade, B1 clade, and B2 clade, top to bottom, respectively. Bottom left panel depicts phylogenetic representations of the F and G clades. More detailed phylogenies can be found in Supplementary Figs. 2 and 3

A plot of genetic distance against time indicates that the evolution of the F and G genes does not follow the assumption of a molecular clock, with R^2^ of 0.005 and 0.03, respectively (Supplementary Fig. 2). It shows distinct trends of evolution corresponding to the different clades. Hence, we analyzed the temporal signal of each “gene: clade” dataset and observed a stronger clock-like trend for all, except for F: A (Fig. 3), which was excluded from the subsequent Bayesian phylogenetic analyses. Their evolutionary rates were consistent with those of other RNA viruses.

The gene-specific clades were found to be evolving at similar evolutionary rates, however, the G clades showed evolutionary rates that were up to 2 times higher than the F clade sequences. Both F clades and G: B2 clade appear to have diverged during the 1980 s, whereas the remaining G clades were estimated to have a most recent common ancestor that is almost 20 years more recent and diverged during the 2000 s (Table 2).

**Table 2:** Evolutionary rates and times of divergence for HMPV F and G genes. Values in parentheses represent 95% Highest Posterior Density (HPD) intervals

Gene	Lineage	Evolutionary rate (substitutions/site/year)	Time of most recent common ancestor
		(95% highest posterior density interval)	(95% highest posterior density interval)
F	B1	2.47 × 10^–3^	1986.29
		(1.87 × 10^–3^—3.13 × 10^–3^)	(1984.42—1987.08)
	B2	1.69 × 10^–3^	1983.54
		(1.30 × 10^–3^—2.11 × 10^–3^)	(1982.19—1983.95)
G	A	4.60 × 10^–3^	2003.84
		(3.76 × 10^–3^—5.54 × 10^–3^)	(2001.53—2005.59)
	B Early	4.09 × 10^–3^	2006.46
		(2.65 × 10^–3^—5.80 × 10^–3^)	(2005.60—2007.24)
	B1	4.99 × 10^–3^	2006.24
		(3.72 × 10^–3^—6.55 × 10^–3^)	(2004.22—2007.84)
	B2	3.83 × 10^–3^	1982.01
		(3.04 × 10^–3^—4.78 × 10^–3^)	(1981.07—1982.38)

### Genetic diversity and demographic dynamics of hMPV

**Fig. 4:**
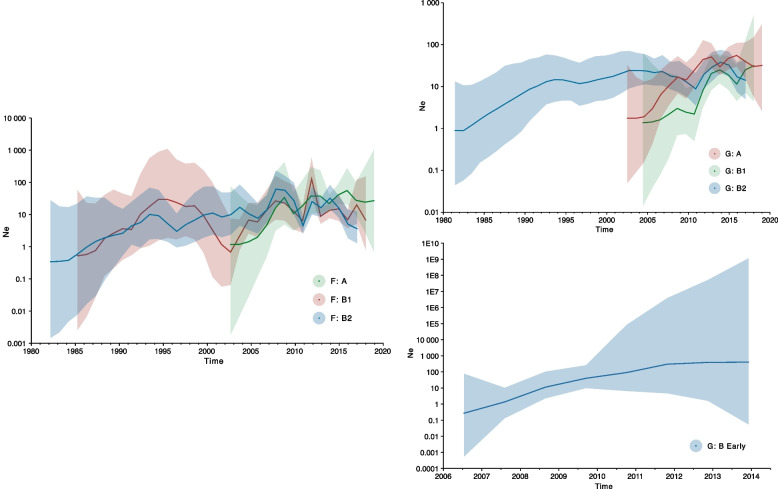
Trends of hMPV’s effective population (N_e_) of F and G genes using the Skygrid model. The shaded portion represents the 95% Bayesian credibility interval, and the solid line is the posterior median. Left panel represents estimates for F gene’s A clade (green), B1 clade (red), and B2 clade (blue). Top right panel represents estimates for G gene’s A clade (red), B1 clade (green), and B2 clade (blue). Bottom right panel depicts the B Early clade (blue) due to the significant differences in the scales of both axes compared to the other clades

Globally, we observed that the hMPV epidemics had a relatively constant viral effective population size (N_e_) for F: A with the most pronounced increase in viral diversity occurring in 2007–2009. Despite the larger uncertainty associated with the demographic inference of F: B1, we can point out one possible event of decrease in viral diversity between 1998–2002, and one period of higher N_e_ in 2011–2012. The demographic patterns for F: B2 also fluctuated over time, with periods of higher viral diversity (2007–2010, 2011–2014) separated by one event where the N_e_ was lower (2010–2011) (Fig. 4—left).

The global viral diversity of G: A increased during 2005–2007 but has remained stable since then (Fig. 4 – top right). The N_e_ of G: B Early was mostly constant with a slight increase in diversity over time (Fig. 4 – bottom right). The demographic patterns of G: B1 were characterized by an increase in population size during the period of 2010–2013 that was mostly unchanged afterwards. The G: B2 clade went through a diversification period from 2010–2014 (Fig. 4 – top right).

### Spatial–temporal patterns of Côte d’Ivoire hMPV epidemics

**Fig. 5:**
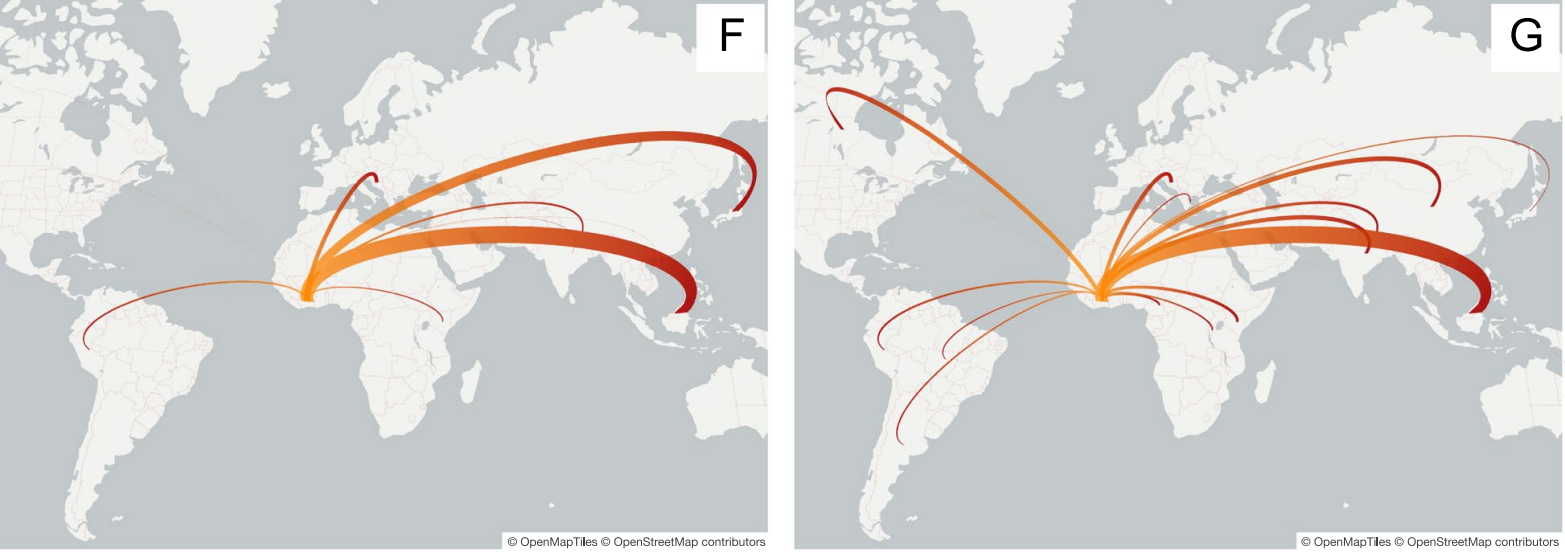
Origin and extent of viral introductions (Markov jumps) of hMPV F gene (left) and G gene (right) viral diversity into *Côte d’Ivoire*

We observed that hMPV epidemics were characterized by multiple introductions into the country throughout the years, originating from different locations (Table 3 and Supplementary Figs. 9–17). F: B1 was introduced once every year (2011, 2013 and 2014) with a most probable origin in Malaysia. F: B2 was introduced twice yearly (2012 and 2013) with origins from Asia and South America (Fig. 5—left). The G clades detected in Côte d’Ivoire during our study period were often estimated to have been introduced as distinct events, with most introductions from Asian countries, but with reasonable posterior probabilities for African and European and North American countries (Fig. 5—right). The low availability of hMPV sequence data from many countries makes it difficult to infer the precise number of viral introductions, but we estimated, conservatively, the timing of introductions that seeded the lineages observed in our 2013–2015 cohort as follows: one introduction (G: B Early) with an estimated TMRCA around the 2008 epidemic season, one introduction (G: B2) around the 2010 season, at least one introduction (F: B1 and G: B2) during the 2011 epidemic season, four introductions (F: B2, G: A and B2) in the 2012, at least five introductions (F: B1 and B2, G: A and B1) in the 2013 season, and one introduction (F: B1) in the 2014 epidemic season. Viruses from multiple epidemic seasons sometimes clustered together, possibly representing persistence of hMPV in Côte d’Ivoire over multiple years. Similarly, some of the times to the most recent common ancestor (TMRCA) were estimated to exceed one year for several viral introductions, based on the time scaled MCC trees (Table 3 and Supplementary Figs. 11–17). However, the low availability of background hMPV sequences from other locations means that we cannot exclude the possibility that similar viruses were re-imported each year from an unsampled location, rather than persisting locally.

**Table 3:** Timing of introductions and respective country of origin for hMPV F and G genes. Values in parentheses represent 95% highest posterior density (HPD) intervals

Gene	Clade	Introductions into Côte d’Ivoire	Country of origin (posterior probability)
F	B1	2014.74 (2014.27—2015.02)	Malaysia (1.00)
		2013.25 (2012.79—2013.59)	Malaysia (0.91)
		2011.37 (2010.67—2012.04)	Malaysia (1.00)
	B2	2013.17 (2012.33—2013.87)	Japan (0.91)
		2013.03 (2011.13—2014.36)	Japan (1.00)
		2012.694 (2011.35—2013.77)	Peru (1.00)
		2012.57 (2011.87—2012.91)	Nepal (0.96)
G	A	2013.38 (2012.06—2014.47)	Malaysia (0.96)
		2012.77 (2012.05—2013.26)	Malaysia (0.63) Croatia (0.31)
	B Early	2008.23 (2007.86—2008.69)	Canada (0.52) India (0.22) China (0.19)
	B1	2013.58 (2012.82—2014.14)	Kenya (0.52) Malaysia (0.47)
	B2	2012.1 (2010.75—2013.18)	Malaysia (0.44)
		2011.69 (2010.61—2012.69)	Malaysia (0.96)
		2010.39 (2009.22—2011.43)	Canada (0.97)

We also compared which clades were dominant each year in Côte d’Ivoire and in the highest probable locations of origin (Malaysia and Croatia), as well as in the USA (Table 4). It appears that the dominant F clade in Côte d’Ivoire in the 2014 epidemic matches the dominant clades circulating in the previous year in Malaysia, with a similar pattern for the 2015 epidemic which matches the dominant clade circulating in 2014 in Malaysia and Croatia. The dominant G clade in the 2014 epidemic in Côte d’Ivoire appears to match the dominant clade circulating in the previous year in the USA. However, these patterns were not clear, which might be an artifact of the limited genetic data available for hMPV worldwide.

**Table 4:** Available sequences per gene for Côte d’Ivoire, USA, Malaysia and Croatia. Dominant clade per year is shown in bold

Gene	Year	Côte d’Ivoire	USA	Malaysia	Croatia
F	2012	0 A	0 A	8 A	**2 A**
		0 B1	0 B1	**23 B1**	1 B1
		0 B2	0 B2	4 B2	**2 B2**
	2013	**6 A**	0 A	6 A	0 A
		2 B1	0 B1	8 B1	**2 B1**
		1 B2	0 B2	**9 B2**	0 B2
	2014	10 A	0 A	2 A	0 A
		3 B1	0 B1	2 B1	**1 B1**
		**19 B2**	0 B2	**5 B1**	0 B2
	2015	4 A	1 A	0 A	0 A
		**16 B1**	0 B1	0 B1	0 B1
		2 B2	**2 B2**	0 B2	0 B2
G	2012	0 A	0 A	6 A	1 A
		0 B Early	0 B Early	2 B Early	**2 B Early**
		0 B1	0 B1	**14 B1**	0 B1
		0 B2	0 B2	3 B2	0 B2
	2013	**6 A**	0 A	5 A	0 A
		0 B Early	0 B Early	0 B Early	0 B Early
		0 B1	0 B1	**7 B1**	**1 B1**
		1 B2	**2 B2**	5 B2	0 B2
	2014	10 A	0 A	2 A	0 A
		0 B Early	0 B Early	0 B Early	0 B Early
		2 B1	0 B1	2 B1	**1 B1**
		**22 B2**	0 B2	**3 B2**	0 B2
	2015	**5 A**	**1 A**	0 A	0 A
		1 B Early	0 B Early	0 B Early	0 B Early
		0 B1	0 B1	0 B1	0 B1
		3 B2	0 B2	0 B2	0 B2

## Discussion and conclusion

The human metapneumovirus (hMPV) imposes a high burden of respiratory disease globally [31]. By sequencing the 113 sequences (38 F and G genes were from the same hMPV strain and 25 unique sequences from F gene and 12 from G gene) of hMPV collected in Côte d’Ivoire, the largest genetic collection of hMPV from West Africa to this date, this study provides insights into the genetic diversity of hMPV in an under-studied region.

The phylogenetic analysis of the F and G genes sequences of hMPV circulating in Côte d’Ivoire during the period from 2013 to 2015 along with a comprehensive background of publicly available genetic sequences, revealed the circulation of two main genetic lineages of hMPV in the country, clade A and clade B. Our phylogenetic analysis (Fig. 2, Table 4) suggests dynamic circulation patterns of hMPV genetic clades in Côte d’Ivoire. While our three-year study period offers a limited window, the observed shifts in genotype prevalence are broadly consistent with studies from other regions that report more definitive annual alternation or replacement of dominant clades [32–34].

We observed hMPV cases year-round, with peaks in Côte d’Ivoire tending to occur during the dry seasons (February–March and July–September), although this was not statistically significant. The seasonality of hMPV varies globally; some studies report year-round circulation [35], similar to our general observation, while others note distinct seasonal patterns often coinciding with influenza and RSV, particularly in temperate regions. Reports also suggest more diverse or less defined seasonality in tropical regions [13]. The absence of strong, statistically significant correlations between hMPV incidence and the climatic factors we analyzed (temperature, humidity, rainfall) suggests that other drivers, or more complex interactions, may influence hMPV seasonality in this tropical setting. Future studies could explore these relationships further using multivariate models incorporating lagged climatic effects or other environmental and population factors, which might reveal more subtle associations.

The majority of the viruses in this study belonged to the B clade. While some earlier studies suggested greater disease severity might be associated with the A group [36], our data did not show a significant association between viral genotype and clinical presentation (*P* = 0.615), with most characterized strains from both lineages A and B being linked to ILI cases. This aligns with other research indicating that the genetic variability of hMPV may not consistently correlate with the clinical severity of the disease [37]. The high ARI burden observed in Côte d’Ivoire is likely multifactorial and not solely attributable to the dominant hMPV genotype.

A slight predominance of males was observed in our study cohort (2062 males to 1837 females; ratio ~ 1.12:1), which may not precisely mirror the sex distribution in the general under-five population of Côte d’Ivoire, potentially reflecting care-seeking behaviors. However, as hMPV transmission is not known to be sex-dependent, we expect that viral mixing occurs irrespective of patient sex and thus this slight imbalance is unlikely to have affected our phylodynamic or transmission pattern inferences [38]. Furthermore, our study may be subject to a geographic selection bias. The national influenza surveillance network is predominantly composed of sentinel sites in major urban and semi-urban centers. Indeed, over 80% of the hMPV-positive cases in our study originated from the coastal cities of Abidjan and San Pedro. This urban focus could limit the generalizability of our findings, as hMPV transmission dynamics and seasonality may differ in rural populations due to variations in population density, mobility patterns, and healthcare access.

We estimated the effective population size of hMPV globally using a nonparametric coalescent model that allows determining the fluctuating diversity of lineages over time [25, 26]. An increase in viral effective population size means that the viral population has more genetic diversity and can respond better to selective pressures, such as host immunity or antiviral drugs. This can lead to faster adaptive evolution and the emergence of new variants. A higher effective population size, as observed in the case of F: A and G gene lineages, allows more mutations to arise and spread, increasing the adaptive potential of the virus, while a lower effective population size, in F: B1 (1998–2002) and F: B2 (2010–2011) at two points in time, reveals a loss of genetic variation and thus a reduction of the adaptive potential of the virus. This may be due to factors like viral population fluctuations or sampling bias [27, 28].

The phylogeographical analysis of the F and G genes allowed us to gain insight into the viral migration patterns characterized by yearly introductions of hMPV viruses into Côte d’Ivoire irrespective of geographical origin. The reconstruction of the spatio-temporal patterns of the G gene were marked by single introductions of the A, B1 and B2 clades from Asian countries and of the B Early clades from Canada and Asian countries. The transmission dynamics of the F gene included multiple introductions of the B1 and B2 clades seeded from Asian countries. These spatial patterns must be interpreted with caution as there are significant gaps in hMPV’s genetic data. Nonetheless, the observed long-distance transmission events and repeated reintroductions into Cote d’Ivoire are characteristic of respiratory viruses [29]. Furthermore, the analysis revealed that hMPV introductions into Cote d’Ivoire occurred after its global establishment around 2005 and most of the introductions originated in Asia [30, 39]. The genetic diversity of hMPV in Asia and its role as a viral source to Cote d’Ivoire and elsewhere is also consistent with previous studies suggesting Asian countries as epicenters of hMPV [40]. However, the process of assembling a comprehensive global dataset for this study underscored the limited availability of hMPV sequences from many regions, particularly across large parts of Africa and other under-resourced areas. These surveillance gaps hinder a complete understanding of global hMPV transmission routes and diversity, limiting the precision of phylogeographic reconstructions, including the exact origins of introductions into Côte d’Ivoire. It also means that we cannot exclude the possibility that similar viruses were re-imported each year from an unsampled location, rather than persisting locally.

The differences in tMRCA estimates for some corresponding F and G gene clades (Table 2) could suggest that distinct evolutionary histories or, speculatively, past recombination events might have shaped the genomic diversity of hMPV, though recombination is generally considered relatively rare in this virus. For instance, the G:B1 clade tMRCA is more recent than might be expected if linked to an F gene lineage B1 with an older tMRCA. However, without specific recombination detection analyses, which were beyond the scope of this work, these observations are merely suggestive. Furthermore, the exclusion of certain variable regions or sequences with unique insertions/deletions such as the G gene duplications reported by Saikusa et al. [21] to maintain alignment quality for phylogenetic inference may introduce biases by potentially removing sequences with functional or evolutionary significance. The impact of such G gene variability on viral fitness or antigenicity warrants further specific investigation. Future studies employing dedicated recombination detection methods would be needed to investigate this possibility more thoroughly for hMPV. The potential for unlinking the evolutionary trajectories of different genomic regions is an important consideration in viral phylodynamics. The phylogeographic modeling of the F and G genes suggests that the annual circulation of hMPV in Cote d’Ivoire is the result of viral strains being imported from other locations. This might also indicate that the virus has limited persistence in the country and that seasonal epidemics are mostly due to viral introductions. The transmission dynamics of HMPV has also been characterized for a tropical location like Peru. Similarly, the authors reconstructed frequent introductions of new HMPV strains into Peru rather than local persistence over time [41]. However, it is crucial to recognize the possible limitations in the present work brought about by sample bias in the worldwide HMPV background dataset that was employed in this investigation. A more equal representation of sequences from all regions, especially those that are currently underrepresented, would be ideal for future research. The A1 clade was not detected in the current analysis which is in line with reports of its global extinction since 2004 [37, 38, 42] and with the premise that older clades are replaced by emerging variants.

The well-established global genomic epidemiology of seasonal influenza virus has allowed recurrent updates of the vaccine formulations according to the circulating variants of the virus [43]. However, the circulation of hMPV in humans is likely underestimated, particularly in Cote d’Ivoire, with instances of deadly spillovers to chimpanzees in the Taï National Park being reported [44]. Our findings on hMPV circulation patterns (Figs. 1 and 2) occur within a broader context of multiple co-circulating respiratory pathogens in Côte d’Ivoire. For instance, during our study period, hMPV peaks often followed influenza peaks. Understanding the specific epidemiology of hMPV, as presented here, is crucial for differentiating its burden from that of other viruses like RSV and influenza, especially given their overlapping clinical presentations and seasonal patterns in tropical regions [13]. This is vital for targeted public health interventions and for accurately assessing the impact of future hMPV-specific control measures. While our study focused on children under five-years-old, a primary group affected by hMPV, it is important to acknowledge that hMPV also causes significant morbidity in older adults and immunocompromised individuals [45, 46]. Future surveillance encompassing a wider age range in Côte d’Ivoire could provide a more complete picture of hMPV epidemiology across the population. Furthermore, continuous genomic and epidemiological surveillance of hMPV in Cote d’Ivoire and globally would be essential for the prompt and precise identification of viral introductions causing epidemics and to inform the implementation of control strategies.

## Supplementary Information

Supplementary Material 1.

## Data Availability

This study generated 20 F gene lineage A, 43 F gene lineage B, 21 G gene lineage A, and 29 G gene lineage B sequences. All hMPV genetic sequences and phylodynamic model parametrizations are available at https://github.com/nidiatrovao/hMPV_IvoryCoast.

## Abbreviations

ARI: Acute Respiratory Infection
BSSVS: Bayesian Stochastic Search Variable Selection
CDC: Centers for Disease Control and Prevention
CHR: Regional Hospital Centre (Centre Hospitalier Régional)
CHU: University Hospital Centre (Centre Hospitalier Universitaire)
CNER: National Ethics Committee for Research (Comité National d’Ethique de la Recherche)
CTMC: Continuous-Time Markov Chain
EnV: Enterovirus
EPI: Expanded Program on Immunization
FSU: Urban Health Unit (Formation Sanitaire Urbaine)
GTR: General Time Reversible
HCoV: Human Coronavirus
hMPV: Human Metapneumovirus
HPD: Highest Posterior Density
hRhV: Human Rhinovirus
ILI: Influenza-Like Illness
MACA: Medical Center of the Civil Prison of Abidjan (Maison d’Arrêt et de Correction d’Abidjan)
MCC: Maximum Clade Credibility
MCMC: Markov Chain Monte Carlo
ML: Maximum Likelihood
Ne: Effective Population Size
nt: Nucleotides
ORF: Open Reading Frame
PIV: Parainfluenza Virus
RASS: Annual Health Situation Report (Rapport Annuel sur la Situation Sanitaire)
RSV: Respiratory Syncytial Virus
RT-PCR: Real-Time Reverse Transcription-Polymerase Chain Reaction
SARI: Severe Acute Respiratory Infection
SODEXAM: Société d’Exploitation et de Développement Aéroportuaire, Aéronautique et Météorologique
tMRCA: Time to the Most Recent Common Ancestor
UTM-RT: Universal Transport Medium
WHO: World Health Organization
