# Supplementary material for: Repeated introductions and widespread transmission of human metapneumovirus in Côte d’Ivoire
Source: BMC Infect Dis. 2025 Sep 2;25:1092. doi: 10.1186/s12879-025-11512-2 (PMC12403408; doi:10.1186/s12879-025-11512-2)
Supplement: Supplementary file 1 — Supplementary Material 1. [file 12879_2025_11512_MOESM1_ESM.docx]

**Supplementary Materials for:**

**Repeated Introductions and Widespread Transmission of Human Metapneumovirus in Côte d’Ivoire**

Hervé A. Kadjo ^1^, Sairah M. Khan ^2#^, Sana Tamim ^3#^, Meriadeg Ar Gouilh ^4^, Marius Adagba^1^, Edgard Adjogoua^1^, Daouda Coulibaly^5^, Astrid Vabret ^4^, Joshua L. Cherry ^2,6^, Martha I. Nelson ^2,6^, Nídia S. Trovão ^2^

1 Department of Epidemic Viruses, Pasteur Institute of Côte d’Ivoire, Abidjan, Côte d’Ivoire

2 Division of International Epidemiology and Population Studies, Fogarty International Center, National Institutes of Health, Bethesda, Maryland, 20892, USA

3 Department of Virology/Immunology, National Institute of Health, Park Road, Chak Shahzad, Islamabad, Pakistan

4 University of Caen Normandy, Dynamicure INSERM UMR 1311, Centre hospital-universitaire (CHU) Caen, Department of Virology, Caen, France

5 Department of epidemiology and disease surveillance, National Institute of Public Hygiene, Abidjan, Côte d’Ivoire

6 Division of Intramural Research, National Library of Medicine, National Institutes of Health, Bethesda, Maryland United States

# Authors contributed equally

**Corresponding author:** Hervé Kadjo ([rvkdjo@yahoo.fr](mailto:rvkdjo@yahoo.fr)), Nídia S. Trovão (nidia.trovao@nih.gov)

**Running title:** hMPV circulation in Côte d’Ivoire.

**Keywords:** Human Metapneumovirus; evolution; disease severity; phylodynamics; acute respiratory infections

***Supplementary Figures***

**
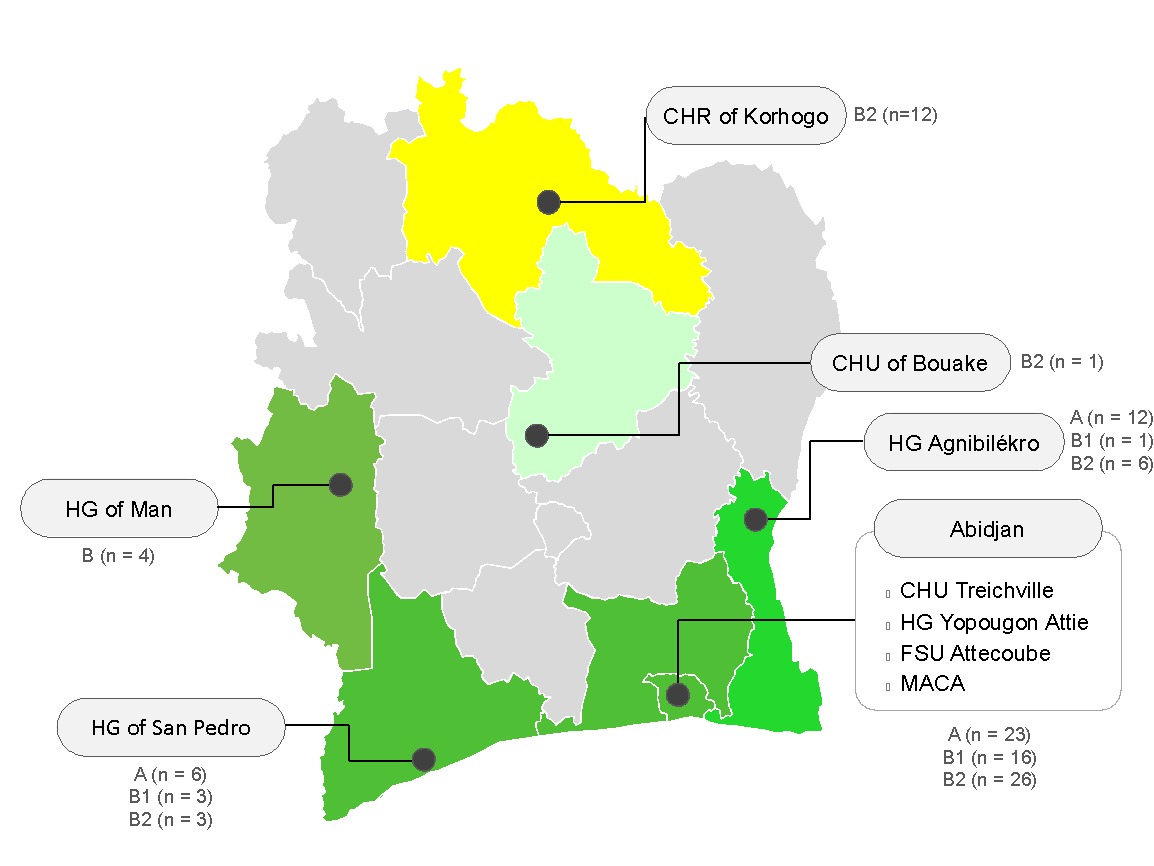
**

**Supplementary Figure S1:** Influenza and respiratory virus circulation surveillance network. Map colors depict vegetation (dark green – forest area; light green – savannah with trees; yellow – savannah). CHU: University Hospital Centre; CHR: Regional Hospital Centre (University Teaching hospital); FSU: Urban Health Unit; MACA: Medical center of the civil prison of Abidjan; HG: General hospital (sub-regional hospital). Source: Respiratory Viruses Unit, Institut Pasteur de Côte d'Ivoire, 2017 Activities Report. Number of samples characterized by lineages are indicated in gray.

**Supplementary Figure S2:** Root-to-tip divergence as a function of sampling time for maximum-likelihood tree of hMPV’s F (top) and G (bottom) genes.

**_
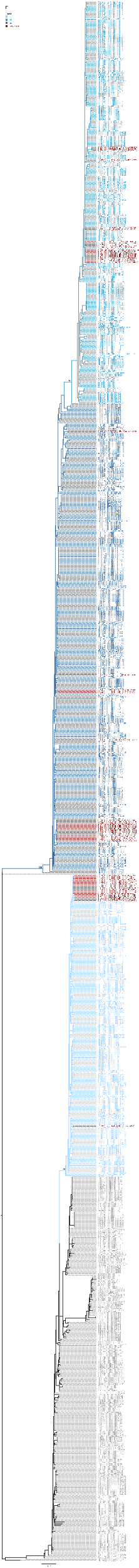
_**

**Supplementary Figure S3:** Maximum likelihood tree of hMPV’s F gene. Phylogenetic tree inferred for F gene sequences collected globally. Sequence tips are colored by clade A, B1, and B2, from light to dark blue, respectively. Sequences collected in Ivory Coast are colored in dark red.

**
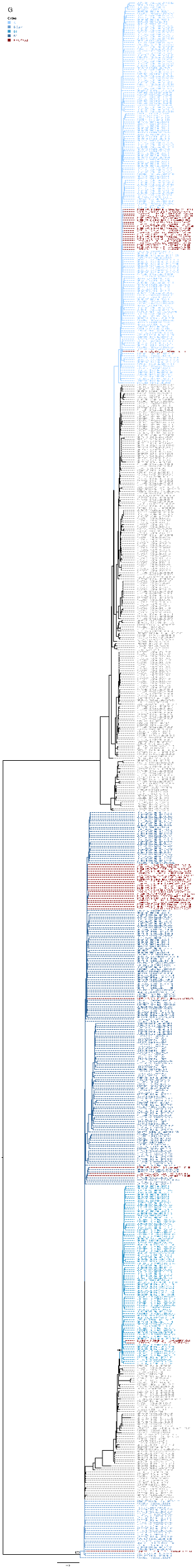
**

**Supplementary Figure S4:** Maximum likelihood tree of hMPV’s G gene. Phylogenetic tree inferred for G gene sequences collected globally. Sequence tips are colored by clade A, B Early, B1, and B2, from light to dark blue, respectively. Sequences collected in Ivory Coast are colored in dark red.

**Supplementary Figure S5:** Number of hMPV samples per location for the F gene dataset. Panels represent counts of F gene sequences for A clade, B1 clade, and B2 clade, top to bottom, respectively.

**Supplementary Figure S6:** Number of hMPV samples per location for the G gene dataset. Panels represent counts for G gene sequences for A clade, B Early clade, B1 clade, and B2 clade, top to bottom, respectively.

**Supplementary Figure S7:** Number of hMPV samples over time for the F gene dataset. Panels represent counts of F gene sequences for A clade, B1 clade, and B2 clade, top to bottom, respectively.

**Supplementary Figure S8:** Number of hMPV samples over time for the G gene dataset. Panels represent counts for G gene sequences for A clade, B Early clade, B1 clade, and B2 clade, top to bottom, respectively.

**Supplementary Figure S9:** Asymmetrical heat maps of hMPV flow between locations for gene F clades. Markov jumps counts measure the expected number of viral movements that occur along the branches of the phylogeny, providing a measure of gene flow. The intensity of the color (red= high; green = low) reflects the percentage of Markov jump counts from location of origin (y-axis) to a destination (x-axis).

**Supplementary Figure S10:** Asymmetrical heat maps of hMPV flow between locations for gene G clades. Markov jumps counts measure the expected number of viral movements that occur along the branches of the phylogeny, providing a measure of gene flow. The intensity of the color (red= high; green = low) reflects the percentage of Markov jump counts from location of origin (y-axis) to a destination (x-axis).

**
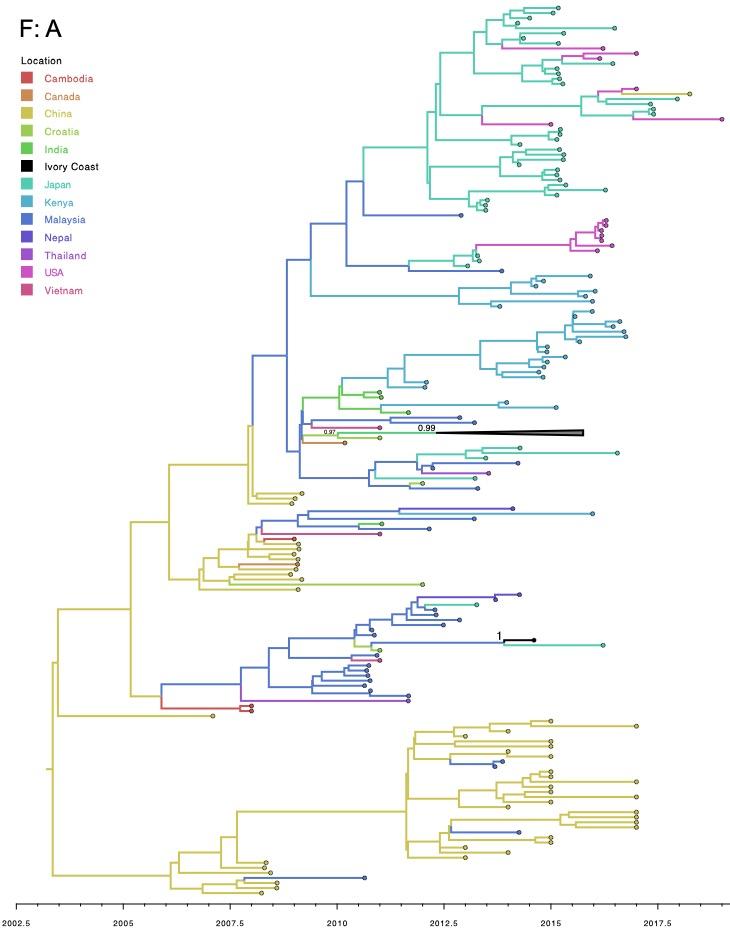
**

**Supplementary Figure S11:** Time-calibrated maximum clade credibility tree inferred for hMPV’s F gene clade A. The shade of the branches and tips indicates the inferred location state at the nodes. The ancestral nodes of Ivory Coast sequences (in black) are annotated with the inferred posterior probability.

**
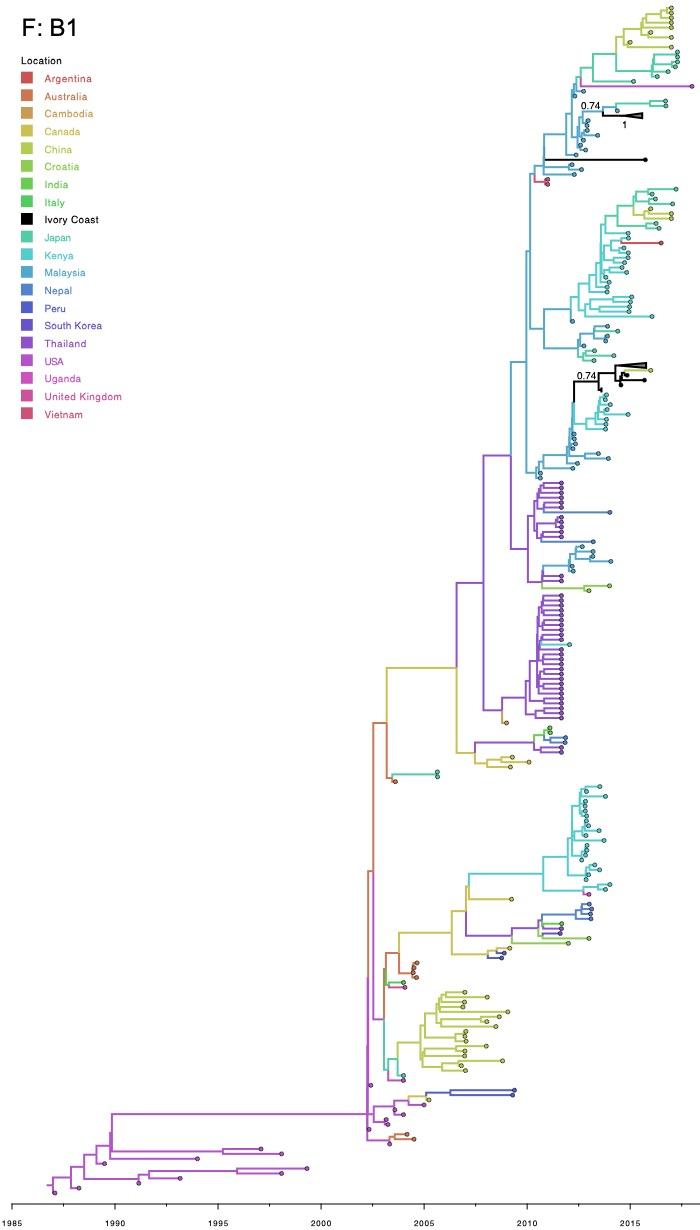
**

**Supplementary Figure S12:** Time-calibrated maximum clade credibility tree inferred for hMPV’s F gene clade B1. The shade of the branches and tips indicates the inferred location state at the nodes. The ancestral nodes of Ivory Coast sequences (in black) are annotated with the inferred posterior probability.

**
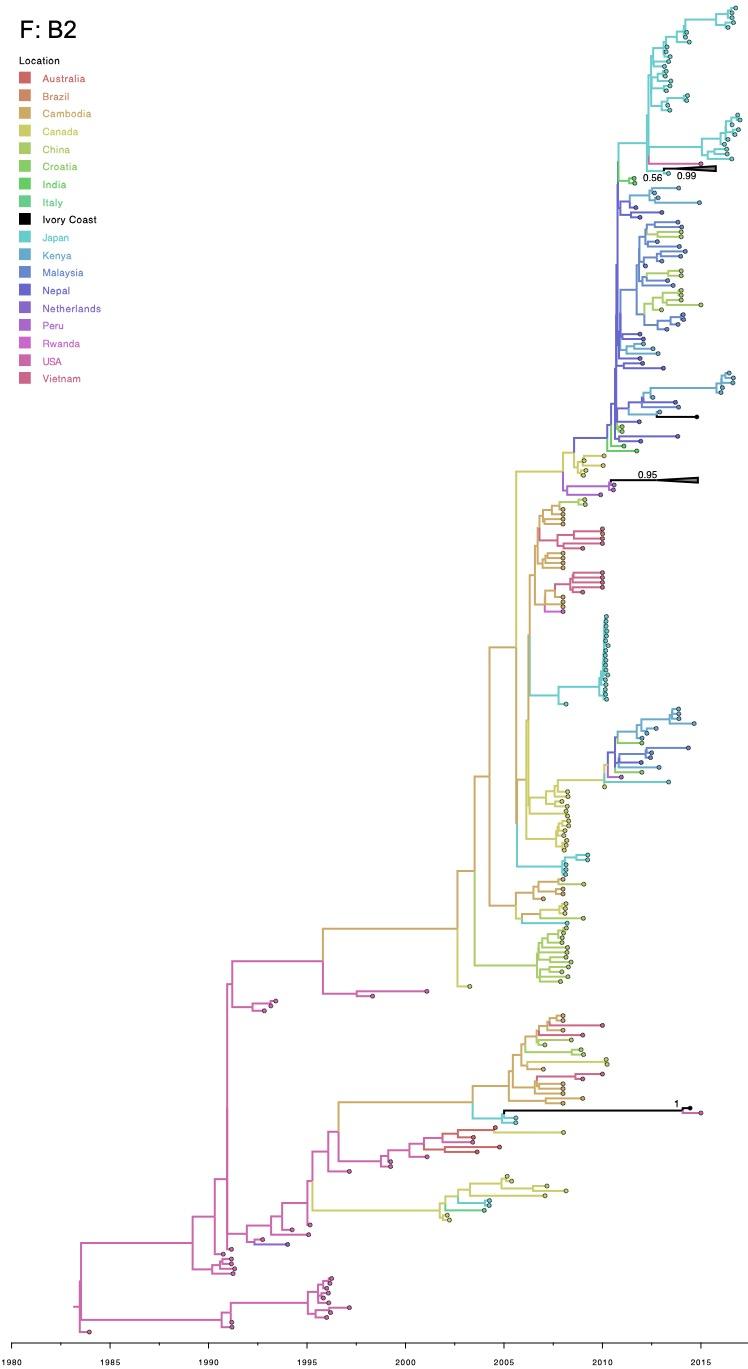
**

**Supplementary Figure S13:** Time-calibrated maximum clade credibility tree inferred for hMPV’s F gene clade B2. The shade of the branches and tips indicates the inferred location state at the nodes. The ancestral nodes of Ivory Coast sequences (in black) are annotated with the inferred posterior probability.

**
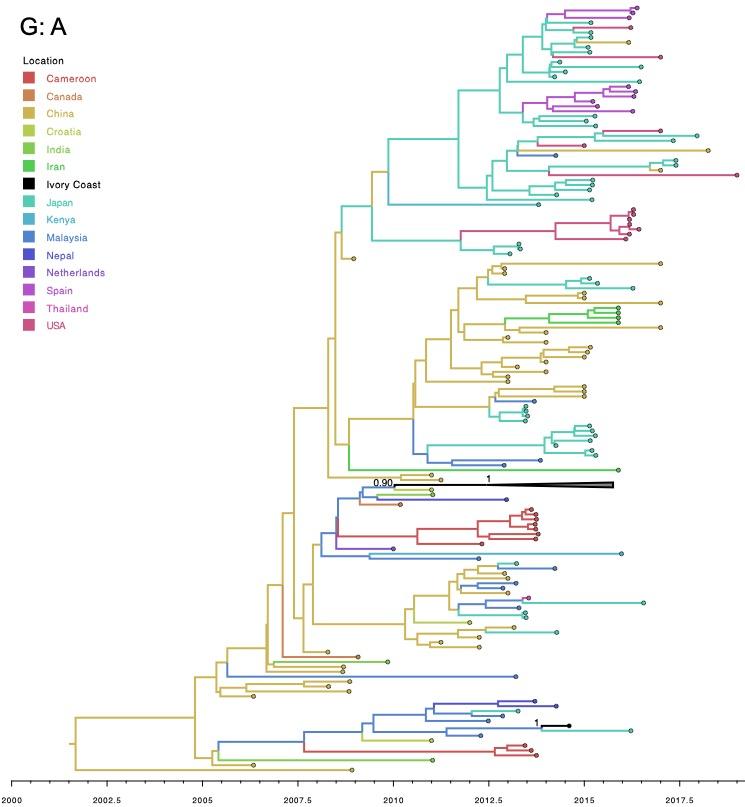
**

**Supplementary Figure S14:** Time-calibrated maximum clade credibility tree inferred for hMPV’s G gene clade A. The shade of the branches and tips indicates the inferred location state at the nodes. The ancestral nodes of Ivory Coast sequences (in black) are annotated with the inferred posterior probability.

**
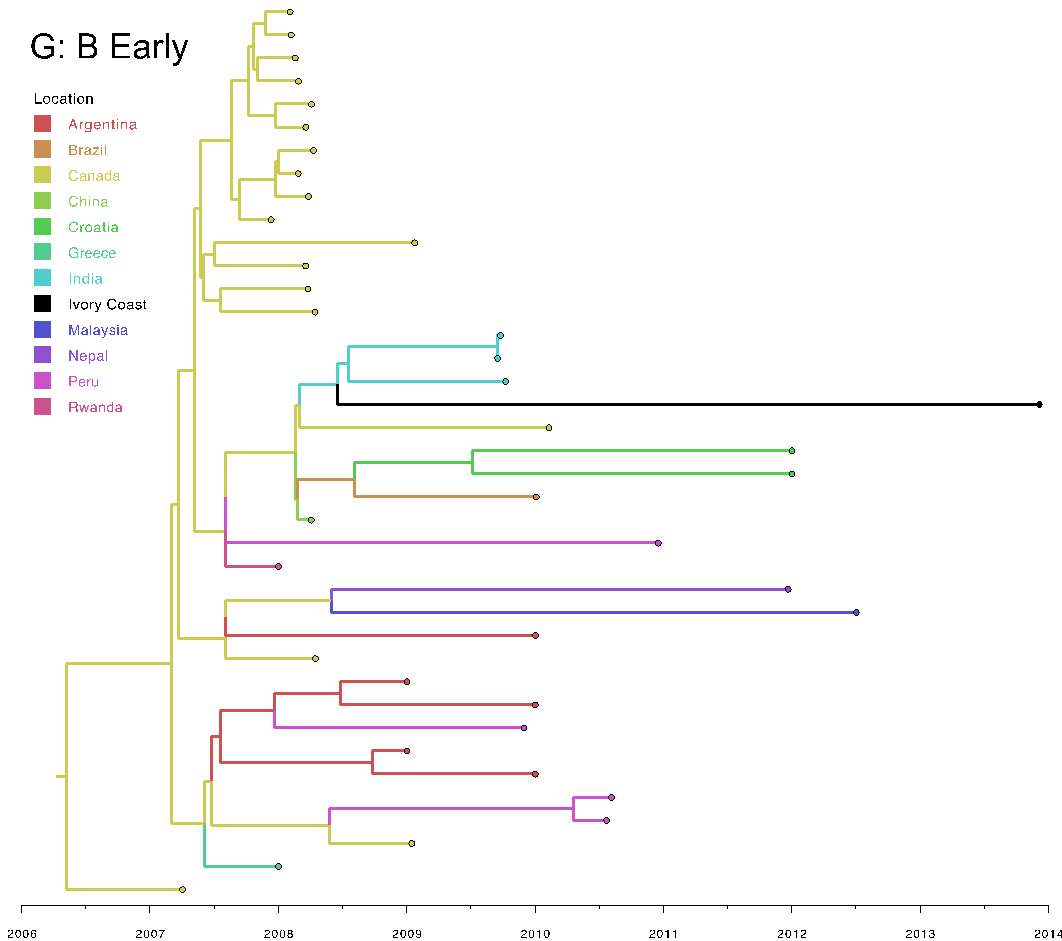
**

**Supplementary Figure S15:** Time-calibrated maximum clade credibility tree inferred for hMPV’s G gene clade B Early. The shade of the branches and tips indicates the inferred location state at the nodes. The ancestral nodes of Ivory Coast sequences (in black) are annotated with the inferred posterior probability.

**
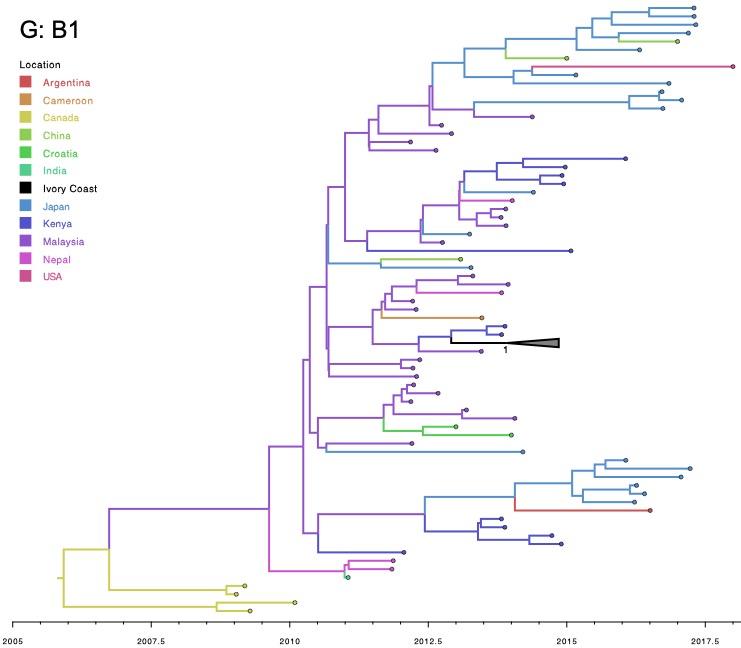
**

**Supplementary Figure S16:** Time-calibrated maximum clade credibility tree inferred for hMPV’s G gene clade B1. The shade of the branches and tips indicates the inferred location state at the nodes. The ancestral nodes of Ivory Coast sequences (in black) are annotated with the inferred posterior probability.

**
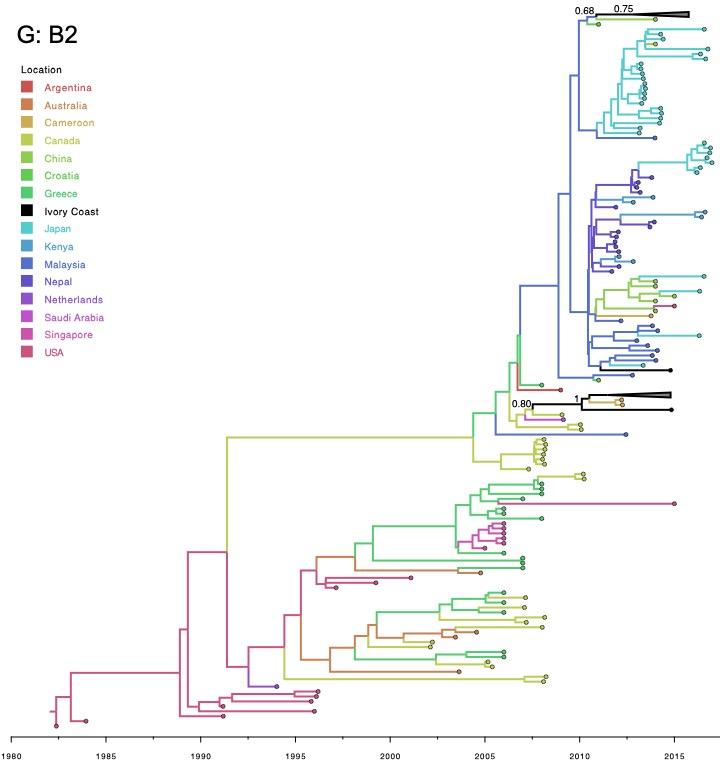
**

**Supplementary Figure S17:** Time-calibrated maximum clade credibility tree inferred for hMPV’s G gene clade B2. The shade of the branches and tips indicates the inferred location state at the nodes. The ancestral nodes of Ivory Coast sequences (in black) are annotated with the inferred posterior probability.

***Supplementary Tables***

**Supplementary Table S1**. Record of data cleaning for “gene: clade” datasets.

| Gene | Clade | Initial count (Background and Côte d’Ivoire) | Duplicates removed | Outliers removed | Final count | Count of sequences from Côte d’Ivoire* |
| --- | --- | --- | --- | --- | --- | --- |
| F | A | 218 | 17 | 7 | 194 | 20 |
|  | B1 | 209 | 32 | 1 | 257 | 21 |
|  | B2 | 324 | 32 | 1 | 291 | 22 |
| G | A | 183 | 3 | 6 | 174 | 21 |
|  | B Early | 42 | 1 | 2 | 39 | 1 |
|  | B1 | 86 | 9 | 3 | 74 | 2 |
|  | B2 | 179 | 6 | 6 | 167 | 26 |
| * Samples preserved throughout the data cleaning process. | | | | | |  |
